# Identification of a six-gene prognostic signature for bladder cancer associated macrophage

**DOI:** 10.3389/fimmu.2022.930352

**Published:** 2022-10-06

**Authors:** Yunzhong Jiang, Xiaowei Qu, Mengzhao Zhang, Lu Zhang, Tao Yang, Minghai Ma, Minxuan Jing, Nan Zhang, Rundong Song, Yuanquan Zhang, Zezhong Yang, Yaodong Zhang, Yuanchun Pu, Jinhai Fan

**Affiliations:** ^1^ Department of Urology, The First Affiliated Hospital of Xi’an Jiaotong University, Xi’an, China; ^2^ Department of Geriatrics, The Yan’an University Xianyang Hospital, Xianyang, China; ^3^ Oncology Research Lab, Key Laboratory of Environment and Genes Related to Diseases, Ministry of Education, Xi’an, China

**Keywords:** tumor associated macrophage, bladder cancer, tumor microenvironment, prognostic model, TAM-related genes

## Abstract

As major components of the tumor microenvironment (TME), tumor-associated macrophages (TAMs) play an exceedingly complicated role in tumor progression and tumorigenesis. However, few studies have reported the specific TAM gene signature in bladder cancer. Herein, this study focused on developing a TAM-related prognostic model in bladder cancer patients based on The Cancer Genome Atlas (TCGA) data. Weighted Gene Co-Expression Network Analysis (WGCNA) was used to identify key genes related to TAM (M2 macrophage). Gene ontology (GO) enrichment and the Kyoto Encyclopedia of Genes and Genomes (KEGG) signaling pathway analysis showed the functional categories of the key genes. Simultaneously, we used the Least Absolute Shrinkage and Selection Operator (LASSO) and univariate and multivariate Cox regressions to establish a TMA-related prognostic model containing six key genes: *TBXAS1, GYPC, HPGDS, GAB3, ADORA3*, and *FOLR2*. Subsequently, single-cell sequencing data downloaded from Gene Expression Omnibus (GEO) suggested that the six genes in the prognostic model were expressed in TAM specifically and may be involved in TAM polarization. In summary, our research uncovered six-TAM related genes that may have an effect on risk stratification in bladder cancer patients and could be regarded as potential TAM-related biomarkers.

## Introduction

Bladder cancer (BC) is the tenth most common cancer in the world, wherein urothelial carcinoma is the most prevalent histological type. Statistically, the worldwide age-standardized incidence rate per 100,000 person/year is 9.5 for men and 2.4 for women (1). In addition, the BC age-standardized mortality rate per 100,000 person/year is 3.3 for men and 0.86 for women (1). Surgery and chemotherapy are the primary treatments for bladder cancer. However, immunotherapy has now increasingly gained scientists’ attention and concern. Several studies verified the benefit of PD-1/PD-L1 checkpoint inhibitors for muscle-invasive and metastatic bladder cancer (2, 3). Macrophages are major component of leukocyte infiltrate into TME. M1 macrophages and M2 macrophages are the common two types (4). Accumulating evidences show that M2 macrophages can promote the tumor proliferation, invasion, and metastasis (5, 6). Therefore, exploring the mechanism of macrophage polarization and reversing the process of polarization may provide a novel treatment for bladder cancer therapy. Furthermore, it is vital to determine TAM-specific biomarkers for targeting immunotherapy. Previous studies have reported few specific markers for TAMs (7, 8). Furthermore, several studies have established TAM-related gene signature (9, 10). However, few studies have demonstrated a TAM-related gene prognostic model in bladder cancer (11). In our study, we attempted to identify key TAM-related genes using WGCNA. We used LASSO and univariate and multivariate Cox regressions to establish a TAM-related prognostic model that enables risk stratification for bladder cancer patients. Additionally, single-cell sequencing data was used to verify the gene signature-specific expression in TAMs. Thus, our study aimed to uncover TAM-related genes and establish a prognostic model, which may have a potential significance for immunotherapy.

## Materials and methods

### Data collection and extraction

The data on gene expression and clinical information were downloaded from TCGA database (http://portal.gdc.cancer.gov/) on March 28, 2022. The former contained 411 bladder tumor tissue and 19 normal bladder tissue samples. The latter included 405 cases. Patients who had missing follow-up data were excluded. Then, 396 patients were included in our research, and we used R 4.0 software to extract the gene expression matrix. In addition, single-cell sequence data were downloaded from Gene Expression Omnibus (GEO) database, with the data being derived from a study focused on chemotherapy-resistant muscle-invasive urothelial bladder cancer (12). Furthermore, we used one primary bladder cancer single-cell data (GSM4307111) to verify the specific expression of our gene signature.

### Fraction of immune cells in TME

CIBERSORT is a powerful bioinformatics tool that can predict the proportion of 22 immune cells infiltrated in TME (13). By using this algorithm, we can observe infiltration of various immune cells in bladder cancer tissues. The results with p<0.05 were preserved. Then, we used ggplot2 R packages to show the TAM proportion in normal and tumor tissues. QuanTIseq is also an algorithm that can quantify tumor immunization according to human RNA-seq data (14). The Immunedeconv R package was used to calculate TAM proportions in the samples by using the quanTIseq algorithm. Furthermore, we used a pie chart to illustrate the different proportion in TAM between normal and tumor samples.

### WGCNA

WGCNA is a method to analyze the gene expression in multiple samples, and it is widely used in studies on correlation between traits and gene association. The WGCNA R package was used to establish a gene co-expression network (15). Genes with an upper 25 median deviation were included and those with no significant expression were excluded. Pearson correlation values were calculated to construct a gene matrix. Then, we chose β=5 as an appropriate soft threshold to construct a scale-free network with high connectivity. Additionally, a topological overlap matrix was produced to estimate the network’s connectivity. Simultaneously, a hierarchical clustering dendrogram of the TOM matrix was established with a minimum size of 30 for every gene dendrogram. We set a cutoff threshold of <0.25 to merge modules with high similarity. Then, the quanTIseq analysis results were combined with the different module eigengenes (MEs). Gene significance (GS) that shows the correlation of gene and immune cell fraction and module membership (MM) that suggests the relationship between genes and gene modules were calculated. Finally, we screened out key genes according to the GS and MM values.

### Functional enrichment analysis and construction of protein-protein interaction network

First, by observing the correlation between gene module and immune cell fraction, we identified TAM-related gene modules. We then screened out representative genes by setting the threshold of GS and MM values. Second, we used the clusterProfiler, ggpolt2 R packages to perform GO enrichment and KEGG signaling pathway analysis for key gene modules. To investigate the interactions between key genes, we performed the PPI network analysis of key genes using String Database (http://string-db.org/) (16). Cytoscape software was used to show the interaction with TAM related genes.

### Construction and validation of gene prognostic model

After screening out TAM-related genes, we combined them with survival data. The 396 patients were randomly divided into training cohorts and validation cohorts at a ratio of 1:1 according to R software. Then, we used the LASSO- Cox regression method to filter genes. LASSO-Cox can prevent model overfitting. It can also refine the model to avoid over-compressing the coefficients. From the analysis, only six genes were filtered out, and we extracted their coefficients to calculate the risk score for every patient. The risk score formula was as follows: risk score = ∑_i_ Coefficient (mRNAi) × Expression (mRNAi). Validation cohorts were used to verify this formula. We divided validation cohorts into high-risk and low-risk groups based on the median risk score in train cohorts. Survival and ROC curve analysis were used to evaluate the model performance and effectiveness. Finally, we regarded the risk score as an independent variable to perform univariate and multivariate Cox regression analysis.

### Expression of key genes in bladder cancer single-cell sequence data

Lee et al. revealed the TME related genes and provided strategic choices to circumvent treatment failure in a chemo-refractory bladder cancer patient by using a single-cell RNA sequence (12). We thus downloaded the single-cell data from the GEO database (www.ncbi.nlm.nih.gov/). Then, we used the Seurat R package to control data quality, reduce data dimension, and perform cell annotation according to the standard proposed by Lee et al. (**Supplementary Figures 2**, **3**). We verified the expression of the gene signature in single-cell data. Finally, the monocle R package was used to detect the differentiation trajectories of cells. We could observe the expression of our key genes in the cell differentiation trajectory.

### Construction of nomogram and correlation between key genes and clinical features

Nomogram is useful for clear demonstration of the prognostic model. We used the regplot function in R software to construct a nomogram which included risk score, risk classification, and clinical features. Then, an ROC curve was plotted to verify the accuracy of our prognostic model. The calibration and DCA curves were also plotted to illustrate the difference between our model and actual observed survival status of patients. Additionally, the correlation between key genes and clinical features was visualized by using the pheatmap R package. Finally, we analyzed survival status and different stages of TCGA patients according to the expression of key genes by using GEPIA (http://gepia.cancer-pku.cn/) (17).

### Statistical analysis

R 4.1.2 software and Graphpad Prism 9 were used to make calculations and statistical analyses. The Wilcoxon rank-sum test was used for comparison of two groups, and the Kruskal-Wallis test was used for comparison of more than two groups. Survival analysis was performed using Kaplan-Meier method with the log-rank test. LASSO and univariate and multivariate Cox regressions were used to construct a prognostic model for bladder cancer patients. The Pearson method was used to analyze the correlation of key genes. P<0.05 was considered statistically significant.

## Results

### Immune cell infiltration in bladder cancer TME

**Figure 1:**
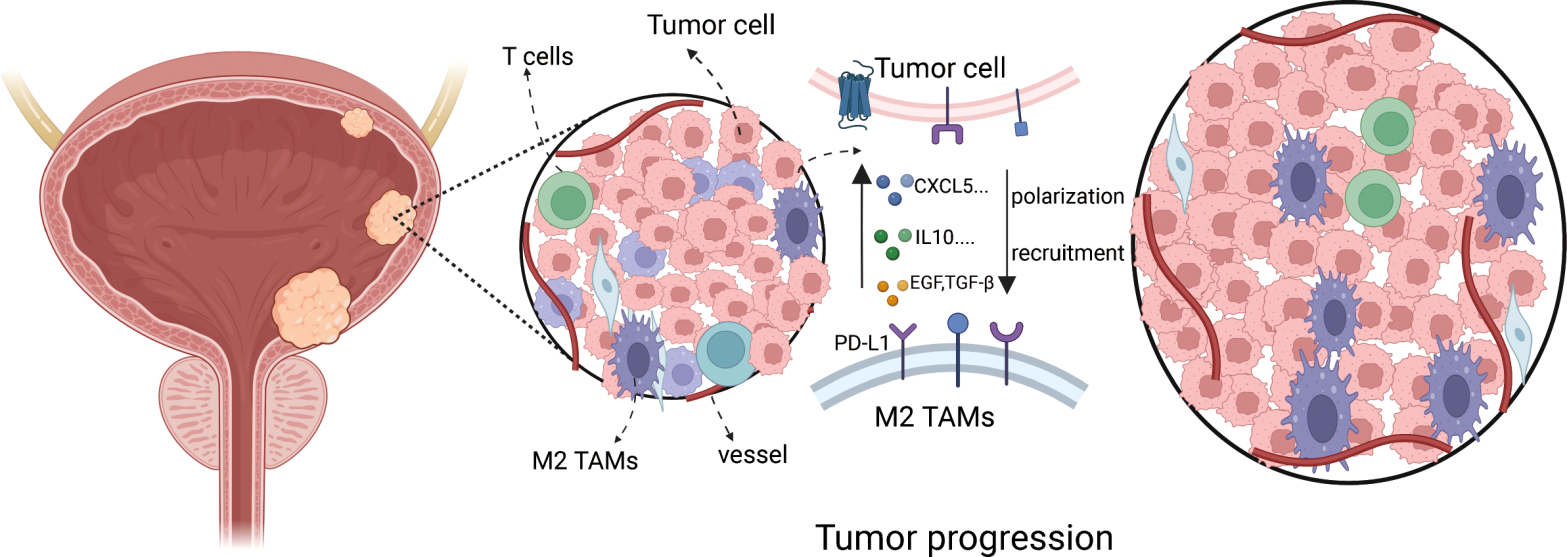
The schematic showing cross-talk between TAMs and bladder cancer cell progression.

**Figure 2:**
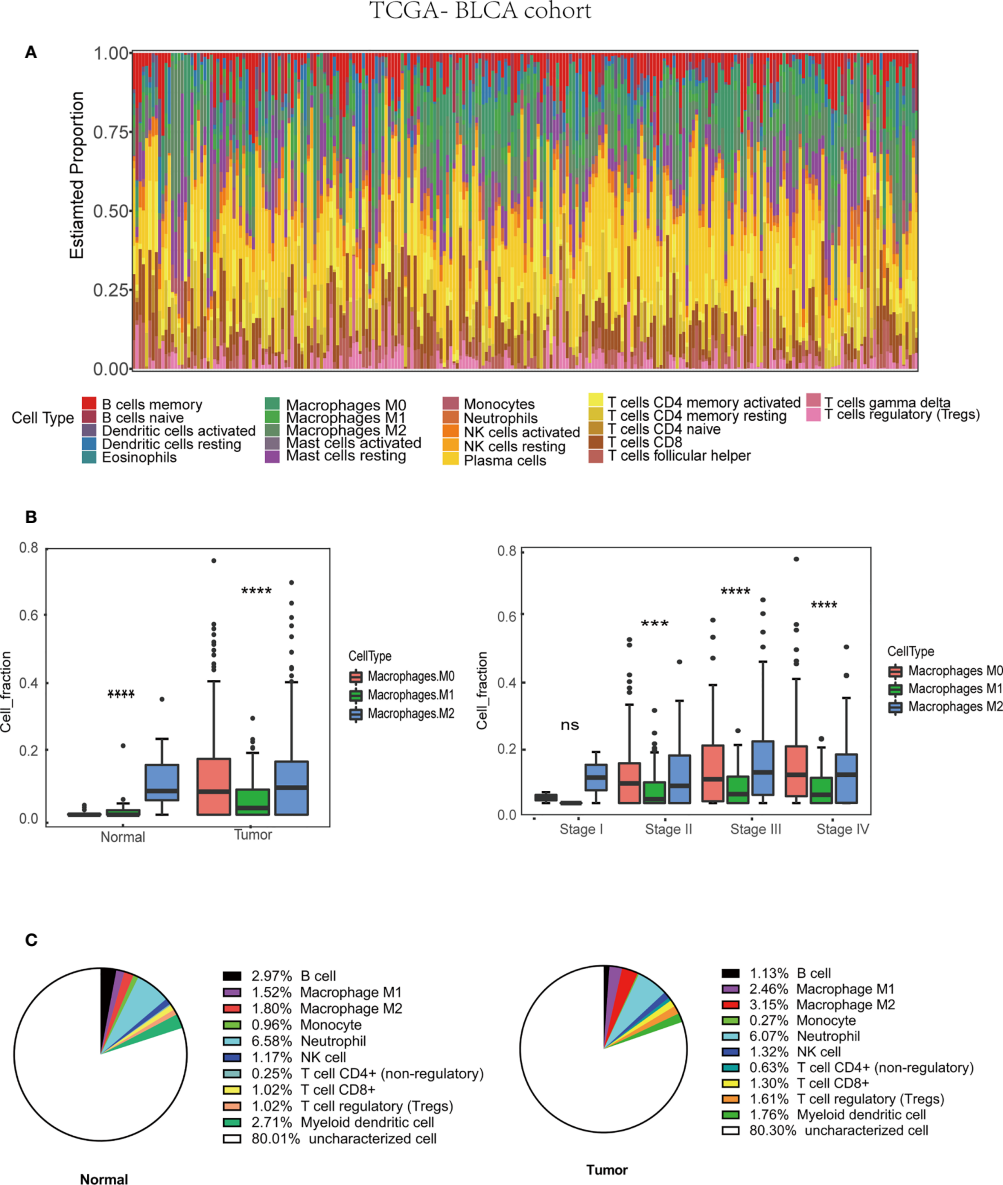
Tumor associated macrophages (TAMs) infiltration in TME based on TCGA-BLCA (Bladder Urothelial Carcinoma) cohort (430 patients). **(A)** Immune cells infiltration in bladder cancer patients. **(B)** M2 macrophages infiltration in various tissue and stage. **(C)** The average M2 macrophage fraction in differ tissue type based on QuanTIseq algorithm.***p < 0.001, ****p < 0.0001.

Many immune cells infiltrate in the bladder cancer TME, such as T cells, B cells, Cancer-associated fibroblasts (CAFs), and TAMs, which play a vital role in tumor progression. The interaction between TAMs and bladder cancer cell is very complicated (**Figure 1**). We used the CIBERSORT method to show the proportion of the immune cells in bladder cancer tissue (**Figure 2A**). Then, the proportion of the macrophage infiltrates in tumors in different tissue and stage was shown in the barplot (**Figure 2B**). We concluded that the proportion of the M2 macrophages was higher than that of M1 macrophages in different tissues. Additionally, we discovered that the proportion of the M2 macrophage increased with increasing tumor stage. Furthermore, quanTIseq was also used to analyze different immune cells’ infiltration in bladder cancer tissue. We calculated the average fraction of various immune cells. The pie chart was used to illustrate the proportion of various immune cells (**Figure 2C**). We found that the proportion of M2 macrophages in normal tissue was lower than that in tumor tissue. From the above results, we can conclude that TAMs, especially M2 macrophages, may play an indispensable role in tumor progression.

### Identification of TAM-related gene modules using WGCNA

**Figure 3:**
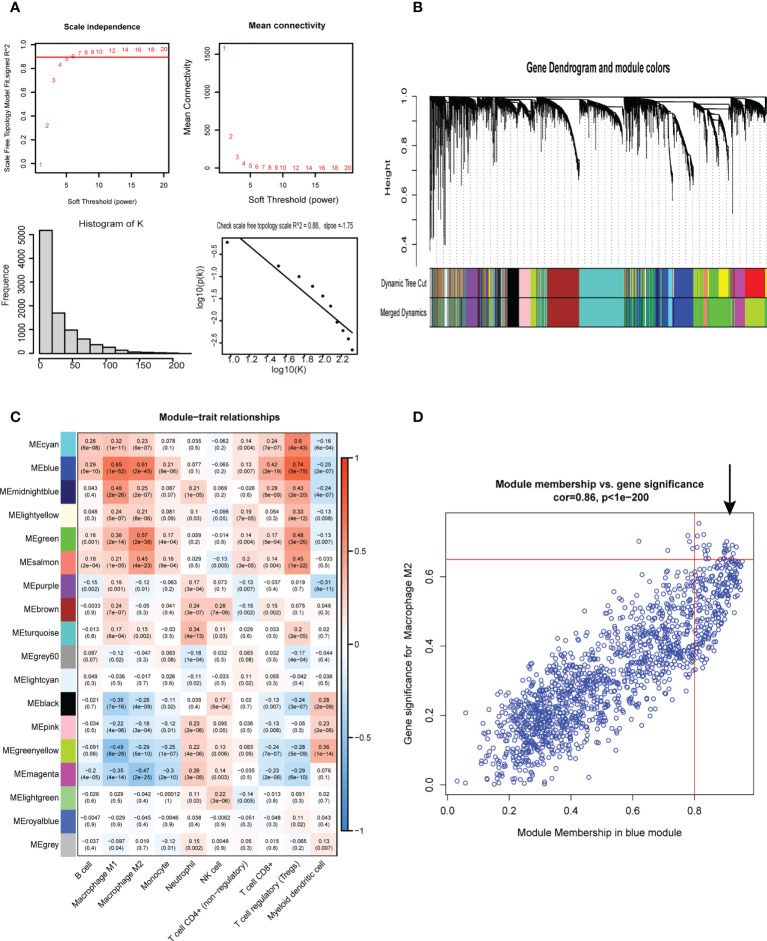
Weighted gene co-expression network analysis. **(A)** Screening for suitable soft thresholds and scale-free network validation. The soft threshold is selected as 5, the distribution curve and network connectivity k, which represented a satisfactory scale-free network. **(B)** The cluster dendrogram with the gene modules and module merging. **(C)**The correlation between gene modules and immune cell fraction. **(D)** The correlation between GS and MM shown in scatter plot in blue modules.

**Figure 4:**
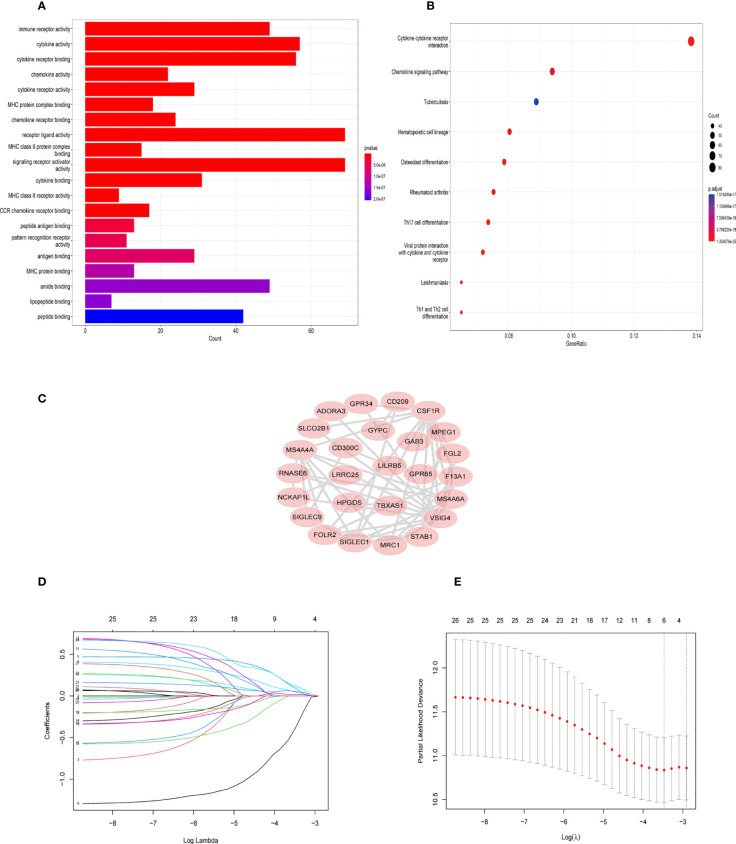
GO and KEGG enrichment analysis in blue module genes and identification of six TAM gene signature. **(A)** Gene ontology (GO) enrichment in blue module genes. **(B)** Kyoto Encyclopedia of Genes and Genomes (KEGG) signaling pathway analysis for blue module genes. **(C)** The network of 26 TAM related genes filtered by the condition (GS>0.65,MM>0.8) **(D)** LASSO regression analysis was used to identify the six genes signature. **(E)** The cross-validation in the LASSO model.

First, 430 patients were clustered using the R software (**Supplementary Figure 1**). Then, we chose β=5 as an appropriate soft threshold to construct a scale-free network (no scale R^2^ = 0.88) (**Figure 3A**). The dynamic cut tree was made after merging similar gene modules (**Figure 3B**). Among the 18 gene module, we found that the blue module had a close relationship with M2 macrophage fraction features (**Figure 3C**). Additionally, we identified the key genes in the blue gene module under the condition of GS >0.65 and MM>0.8 (**Figure 3D**). Furthermore, we performed a GO enrichment and KEGG signaling pathway analysis for the blue module. The genes in the blue model were correlated to immune receptor activity, cytokine activity, MHC protein binding, among others (**Figure 4A**). The KEGG pathway analysis suggested that the genes were involved in the chemokine signaling pathway (**Figure 4B**).

### Construction of the PPI network and six-gene prognostic model

Twenty-six key genes were screened out under the conditions: GS >0.65 and MM>0.8. Then, we performed the protein-protein interaction (PPI) network analysis of 26 genes using String Database (http://string-db.org/) (16). The Cytoscape software was used to visualize the above results (**Figure 4C**). In addition, we used Lasso-Cox regression to filter genes (**Figures 4D, E**). From the analysis, only six genes were screened out, namely *TBXAS1, GYPC, GAB3, HPGDS, ADORA3*, and *FOLR2*. Their coefficients were 0.3703, 0.1809, -1.299, 0.122, 0.3304, and 0.0236, respectively. Furthermore, we calculated the risk score for 396 patients according to the formula:


risk score =TBXAS1*0.3703+GYPC*0.1809+GAB3*−1.299+HPGDS*0.122+ADORA3*0.3304+FOLR2*0.0236

### Evaluation of six-gene signature

**Figure 5:**
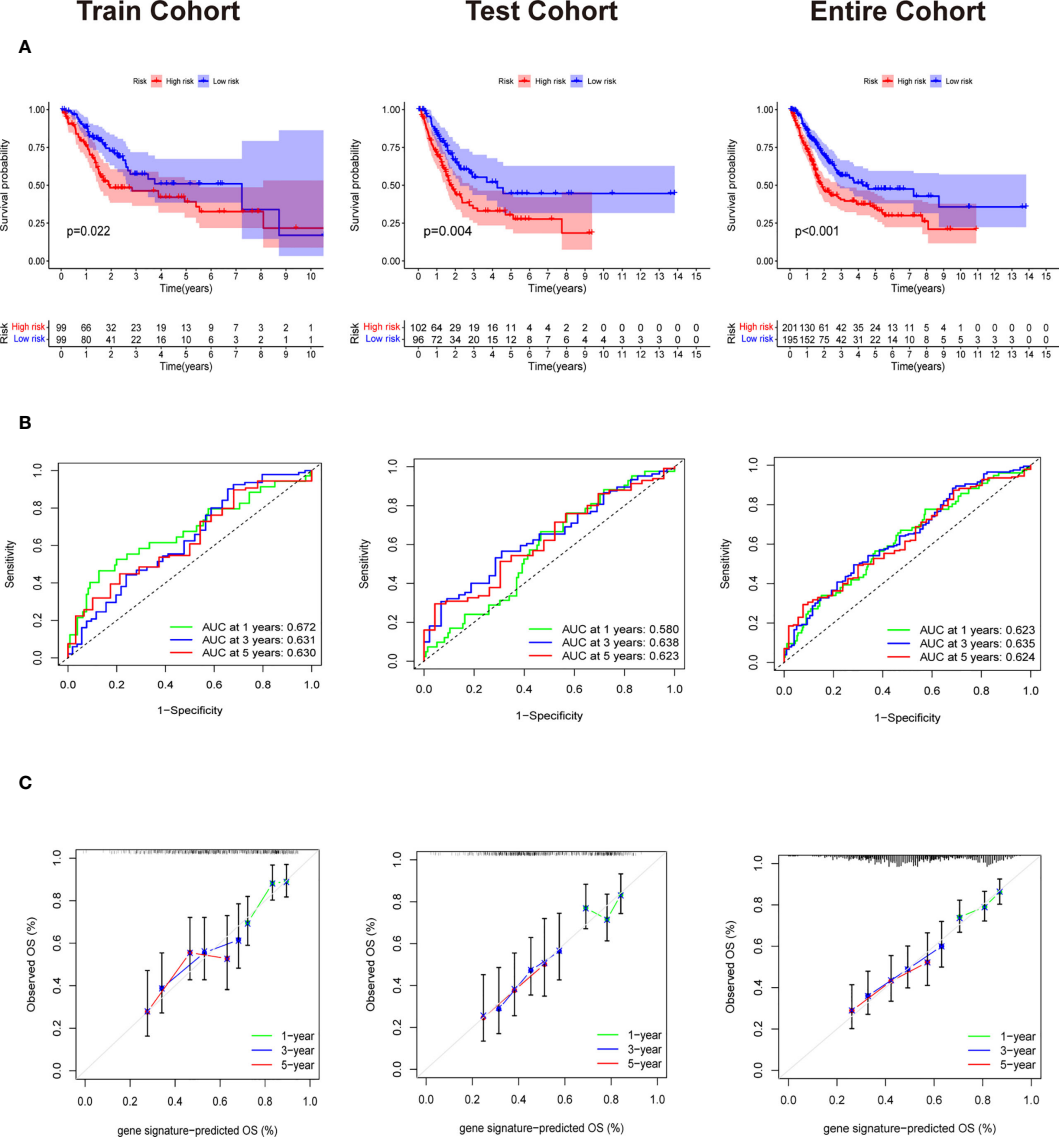
Validation of Evaluation of Six gene signature. **(A)** The Survival curve based on Kaplan Meier with log-rank test in train cohort,test cohort and entire cohort. **(B)** Time-dependent ROC curve means the accuracy of our model in predicting the 1 year, 3 year and 5 year survival rate. **(C)** Calibration curve that predicted 1 year, 3 year and 5 year survival probability.

**Figure 6:**
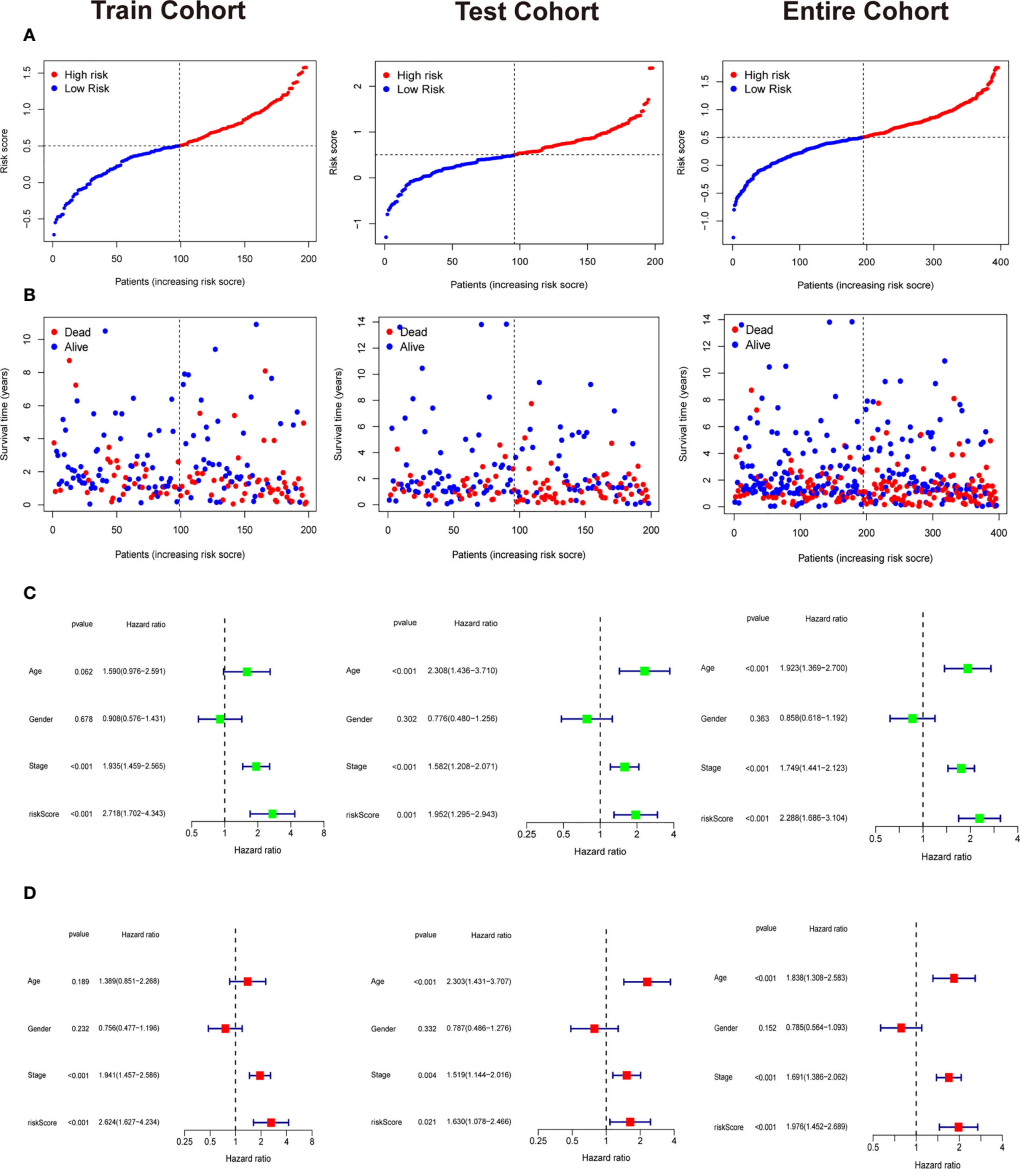
Independent prognostic analysis and the correlation between survival status and riskscore. **(A)** The riskscore curve in train cohort, test cohort, and entire cohort. **(B)** Patients’ status as riskscore increases in train cohort, test cohort, and entire cohort. **(C)** Univariate Cox analysis in train cohort, test cohort, and entire cohort. **(D)** Multivariate Cox analysis in in train cohort, test cohort, and entire cohort.

Patients in training cohorts were divided into high-risk and low-risk groups. Kaplan-Meier with log-rank test method was used to observe the difference between the two groups. We concluded that the patients in the low-risk group had a longer survival time than that in the high-risk group (**Figure 5A**). Moreover, a time-dependent ROC and calibration curves were drawn to verify our model’s accuracy (**Figures 5B, C**). The area under ROC curve in the train was nearly 0.6, and the calibration curve demonstrated the difference between patient actual survival status and predictive status in 1, 3, 5 years. The risk curve and survival status plot are shown in **Figures 6A, B**. It was demonstrated that the patient’s risk had a positive correlation with their risk score. Finally, we regraded risk score as an independent variable and combined it with other clinical features to perform univariate and multivariate Cox analyses (**Figures 6C, D**). We concluded that the risk score based on the six genes and tumor stage are independent prognostic factors in patients from TCGA cohort.

### The expression of key genes in the ScRNA sequence data

**Figure 7:**
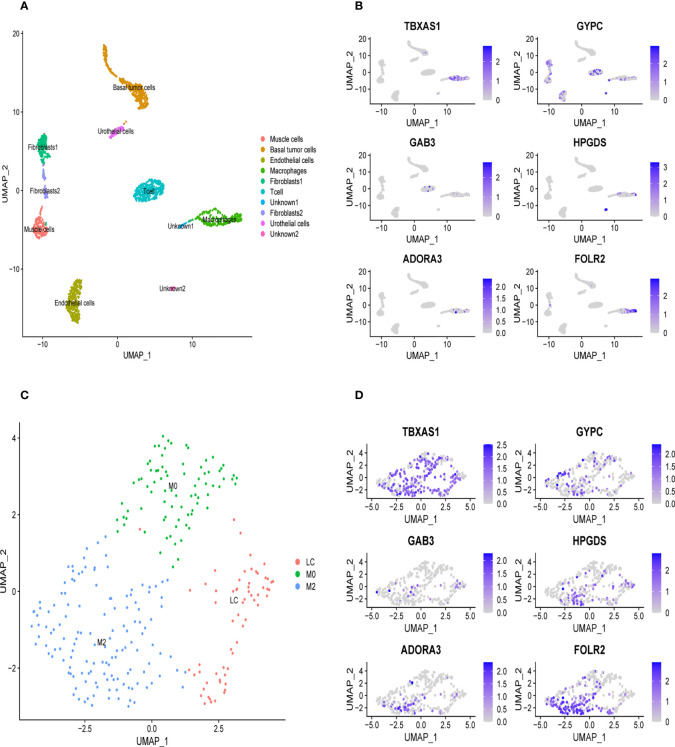
Validation of the specific expression level of six genes in single cell sequence data based on GSE145137 **(A)** Umap plots of single cell sequence data in GSE145137 **(B)** The expression level of six genes in macrophages. **(C)** Macrophage is subdivided into three clusters. **(D)** The expression level of six genes in M2 macrophage.

**Figure 8:**
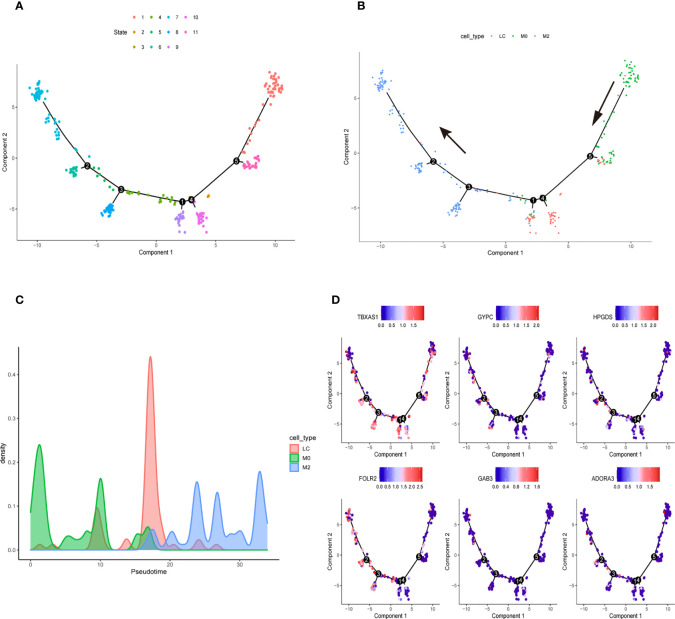
Cell Trajectory Reconstruction and Pseudo-temporal Analysis **(A)** Construction of cell trajectory by cell state **(B)** Construction of cell trajectory by cell type. **(C)** Cell density plots along the time axis. **(D)** The expression level of six genes in the macrophage trajectory plots.

We downloaded the single-cell data from Gene Expression Omnibus (GEO)(www.ncbi.nlm.nih.gov/). Then, we used the Seurat R package to cluster cells and make annotations according to the standard mentioned above (**Figure 7A**). The expression of the six key genes was visualized by the umap function in the Seurat package (**Figure 7B**). Furthermore, we extracted the data sequence of the macrophage and divided it into three clusters. The top ten differential genes are shown by the heatmap (**Supplementary Figure 4**). Then, we annotated cells according to well-known macrophage markers (**Figure 7C**). The six genes’ expression is also shown using umap. We observed that the key genes were highly expressed in M2 macrophages (**Figure 7D**). Finally, we used the monocle R package to show the cell differentiation trajectory (**Figures 8A, B**). The cell density plot in chronological order is shown in **Figure 8C**. The six genes’ expression was correlated with the differentiation of M0 macrophages into M2 macrophages (**Figure 8D**). The change in expression of the six genes with cell state is also shown in **Supplementary Figure 5**.

### Construction and validation of nomogram

**Figure 9:**
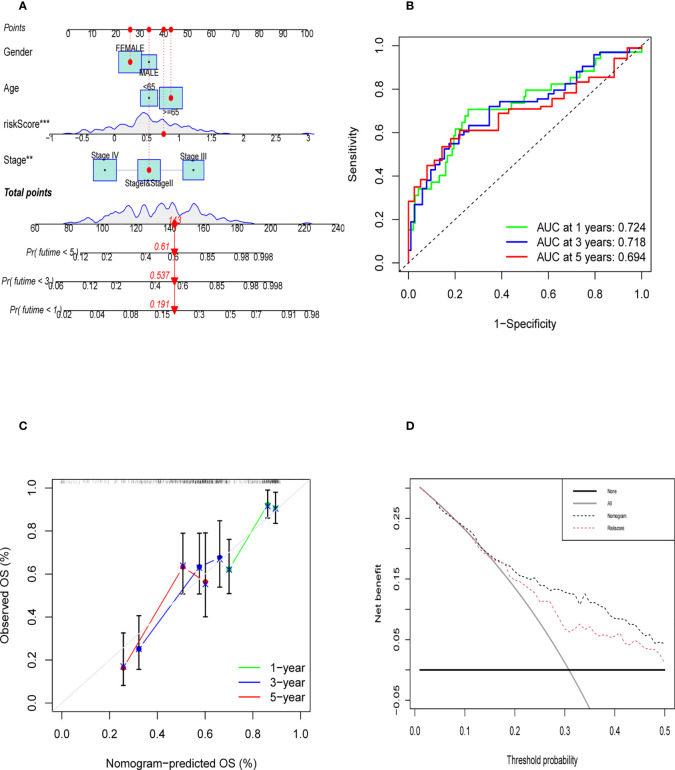
Evaluation of Validation of Nomogram. **(A)** The nomogram was established to predict the prognosis of patients. **(B)** The time-dependent ROC curve was shown the accuracy of our nomogram **(C)** The calibration curve of nomogram that predict the 1,3,5 year survival rate. **(D)** DCA curve was shown that the nomogram was better than riskscore on predicting survival rate of patients.

Nomogram was established to visualize our results of multivariate Cox (**Figure 9A**). It also provided a reference model to predict the 1-year, 3-year, and 5-year survival rates of bladder cancer patients. Then, we plotted the ROC curve to verify the accuracy of our prediction model. The area under the ROC curve was approximately 0.7 (**Figure 9B**). The calibration curve was also plotted to observe the difference between the survival rate predicted by the nomogram and the actual survival rate (**Figure 9C**). At last, we used the DCA curve to demonstrate whether our nomogram can benefit in predicting the survival rate compared to risk score alone (**Figure 9D**). The survival curve, graph of clinical stages, and other clinical features heatmap of patients in TCGA cohort based on the expression level of six genes were shown in **Supplementary Figures 6A–C**, which demonstrated that the expression level of six genes had positive correlation with clinical stage, pathological grade and other clinical features. Furthermore, there was a negative correlation between the expression level of six genes and overall survival of patients. The relative heatmap illustrated that our gene signature had a close correlation with common M2 macrophage biomarkers (**Supplementary Figures 6D**, **7**).

## Discussion

Recently, an increasing number of studies have focused on tumor microenvironment (TME) (18, 19). Tumor associated macrophages (TAMs) are major component in TME. M2 TAMs play an important role in promoting tumor growth. Therefore, it is imperative to find TAM-specific markers for cancer treatment. Cassetta et al. constructed a TAM-related gene signature in breast cancer, which was highly enriched in aggressive breast cancer subtypes (20). Xu et al. also establish an M2 macrophage-related gene prognostic model in pancreatic ductal adenocarcinoma (21). However, a few studies have constructed a TAM-related gene prognostic model in bladder cancer. In our study, we used the WGCNA method to identify TAM (M2 macrophage)-related genes. Simultaneously, LASSO-Cox was used to filter genes to construct the six-gene signature containing: *TBXAS1, GAB3, HPGDS, GYPC, FOLR2*, and *ADORA3*. Fernando et al. performed the transcriptome profiling that revealed the key genes associated with human monocyte-to macrophage differentiation and polarization activation (22). Furthermore, they also summarized the well-known M2 macrophage makers. In our study, not only did we screen out specific TAM-related genes, but also constructed a six-gene prognostic model. Additionally, we calculated the risk score for TCGA bladder cancer patients based on our gene signature.

Wang et al. also used the weighted gene co-expression network analysis (WGCNA) to discover the TAM-related genes in bladder cancer (23). In our study, we constructed a TAM-related gene prognostic model and validated it using bladder cancer single-cell sequence data, making our study different from their study. Lee et al. used their own single-cell RNA sequence to reveal the TME related genes and provide strategic choices to circumvent treatment failure in a chemo-refractory bladder cancer patient (12).To verify the expression of our six TAM-related genes, we obtained the single-cell sequence from the above-mentioned study, which was from a primary bladder cancer sample.

Additionally, we also discovered that our six genes may be correlated with the process of TAM polarization. *TBXAS1*, which is known as Thromboxane A synthase 1 encodes the member of the cytochrome P450 superfamily of enzymes which plays an important role in drug metabolism and synthesis of cholesterol, steroids and other lipids. It was reported that *TBXAS1* protein had strong correlation with pathophysiological processes including hemostasis, cardiovascular disease, and stroke (24). Furthermore, it was also reported that the *TBXAS1* protein was significantly enriched in TAM exosomes, and its expression was correlated with tumor progression (25). *GAB3*, which is known as Growth Factor Receptor Bound Protein 2-Associated Protein is involved in several growth factors and cytokine signaling pathway (26). Furthermore, a study performed by Wolf et al. (27) suggested that *GAB3* could facilitate macrophage differentiation. *GAB3* was also reported to promote tumor progression, particularly in ovarian cancer, colorectal cancer, and glioma (28–30). *GYPC*, which is known as Glycophorin C plays a vital role in regulating the mechanical stability of red cells (31). Guo et al. indicated that a three-gene prognostic model, which contained the *GYPC* gene, can predict the survival rate in ovarian cancer (32). *HPGDS* (Hematopoietic prostaglandin D synthase) is an integral membrane glycoprotein, which is related to various diseases including blood system diseases and malaria (33). Li et al. also constructed six-gene signature, which included *HPGDS*, and this signature was found to be associated with the diagnosis and prognosis of lung adenocarcinoma (LUAD) patients (34). *ADORA3* (Adenosine A3 Receptor) which encodes a protein that belongs to the family of adenosine receptors mediates a sustained cardioprotective function during cardiac ischemia (35). Additionally, it was also reported that *ADORA3* was a potential target for antibody-based therapy in p53 mutated lung tumors (36). *FOLR2* (Folate Receptor Beta) is protein coding gene which could bind to folate and reduce folic acid derivatives. Neural Tube Defects and folate malabsorption were reported to have correlation with *FOLR2* (37). Meanwhile, *FOLR2* is also a common M2 macrophage biomaker as reported by many studies (38, 39).

Few studies had reported that the six genes (*TBXAS1, GYPC, HPGDS, GAB3, ADORA3, and FOLR2*) had strong correlation with bladder cancer. However, in our research, we discovered that the six genes were specifically expressed in M2 macrophage in bladder cancer patients. In addition, there was a negative correlation between the expression level of six genes and overall survival of bladder patients in TCGA. Meanwhile, we also found that the expression level of six genes had positive correlation with the expression of CD163 which was the well-known biomakers of TAMs. Furthermore, the bladder cancer patients in TCGA were divided into two groups according to the risk score calculated by our six gene signature. We could clearly find that the high risk patients had poor prognosis compared to low risk patients. In conclusion, we speculated that our six genes could promote bladder cancer progression by affecting bladder cancer associated macrophages polarization. And the uncovered six TAM-related genes may have a meaningful guide on risk stratification in bladder cancer patients. However, our research results are only based on TCGA database, multiple clinical studies and biology experiments are needed verify our results.

## Conclusion

In summary, we used the WGCNA method to screen out TAM-related genes in bladder cancer. Lasso regression, univariate, and multivariate Cox analyses were used to construct a six gene prognostic model for bladder cancer patients. The single-cell sequence data verified the accuracy of the six key genes expressed in M2 macrophage. Cell trajectories analysis suggested that these six genes may be involved in the process of TAM polarization. Hopefully, our six-gene signature prognostic model may provide important information for future studies on prognosis and treatment of bladder cancer.

## Data availability statement

Publicly available datasets were analyzed in this study. This data can be found here: TCGA(http://portal.gdc.cancer.gov/) and GEO (www.ncbi.nlm.nih.gov/) under the accession number GSE145137.

## Author contributions

YJ and MZ had full access to all the data in the study and takes responsibility for the integrity of the data and the accuracy of the data analysis. YJ used R software to perform statistical analysis and draft this manuscript. TY, RS, YQZ, YP, YDZ, ZY, and MJ take part in the process of acquisition of data, analysis and interpretation of data. All authors contributed to the article and approved the submitted version.

## Acknowledgments

We would like to sincerely appreciate all colleagues from the Department of Urology, The First Affiliated Hospital of Xi’an Jiaotong University, for their support. **Figure 1** was created with Biorender.com

## Conflict of interest

The authors declare that the research was conducted in the absence of any commercial or financial relationships that could be construed as a potential conflict of interest.

## Publisher’s note

All claims expressed in this article are solely those of the authors and do not necessarily represent those of their affiliated organizations, or those of the publisher, the editors and the reviewers. Any product that may be evaluated in this article, or claim that may be made by its manufacturer, is not guaranteed or endorsed by the publisher.

## Supplementary material

Supplementary Figure 1 The clustering of 430 patients in TCGA database.

Supplementary Figure 2 Data filtration and quality control in data downloaded from GEO (GSE145137). **(A)** The number of RNA expressed in various cells and the percentage of mitochondrial genes. **(B)** The correlation of the counts of RNA expression and percentage of mitochondrial genes. The number of genes is positively related to the number of RNA.

Supplementary Figure 3 Dimensionality reduction by PCA analysis **(A)** PCA results shown in scatter plot. **(B)** The ElbowPlot shows that results of PCA analysis. **(C)** The top 10 differentially expressed genes from PCA1 to PCA15.

Supplementary Figure 4 Clustering and pseudo-chronological analysis of macrophages. **(A)** Umap plots of macrophage classification **(B)** The top 10 differentially expressed genes in three clusters. **(C)** Cell trajectory map established by pseudotime value. **(D)** The heatmap shows that the expression level of six genes in pseudo-chronological analysis.

Supplementary Figure 5 The expression level of six genes with the changes in cell state. **(A)** The distribution of six genes in different cell state **(B)** The violin plot of the expression level of six genes in different cell state. **(C)** Line chart of the expression of six gene in different cell state.

Supplementary Figure 6 The correlation between the expression level of six genes and clinical features. **(A)** The Kaplan Merier survival curve of TCGA-patients based on expression level of six genes. **(B)** The correlation between tumor stage and the expression level of six genes. **(C)** The heatmap shows the correlation between the expression of six gene and clinical features. **(D)** Heatmap shows that the correlation between common M2 biomakers and six gene signature.

Supplementary Figure 7 The correlation between the expression level of six genes and the expression level of CD163.
