# Supplementary figures and images for: Identification of a six-gene prognostic signature for bladder cancer associated macrophage
Source: Front Immunol. 2022 Oct 6;13:930352. doi: 10.3389/fimmu.2022.930352 (PMC9582252; doi:10.3389/fimmu.2022.930352)

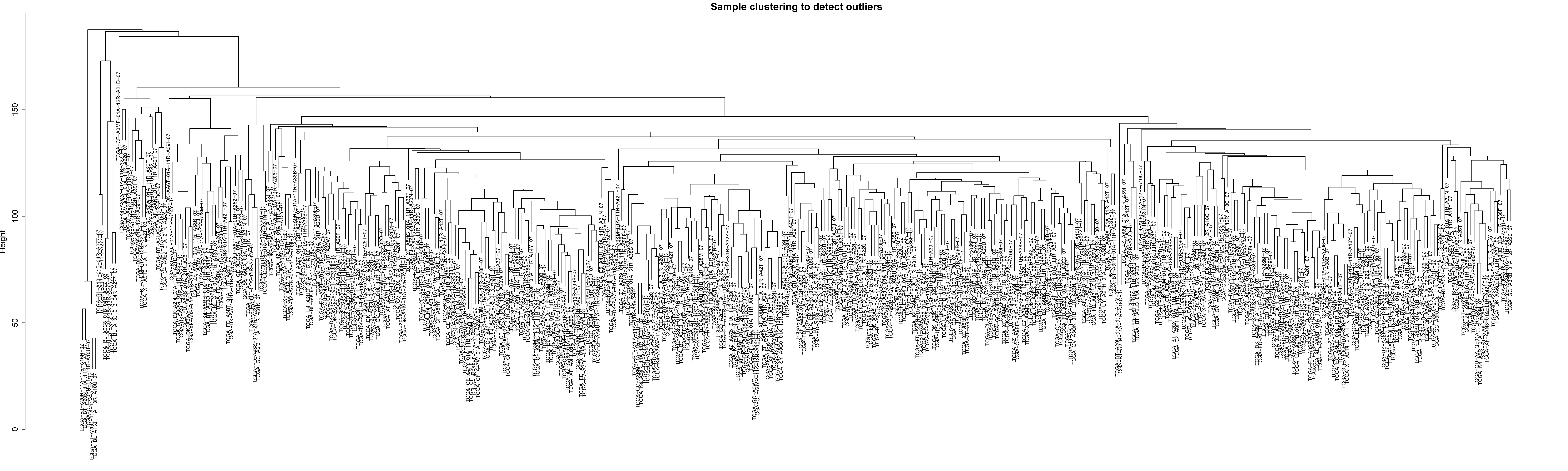

Supplement: Supplementary Figure 1 — The clustering of 430 patients in TCGA database. [file Image_1.tif]

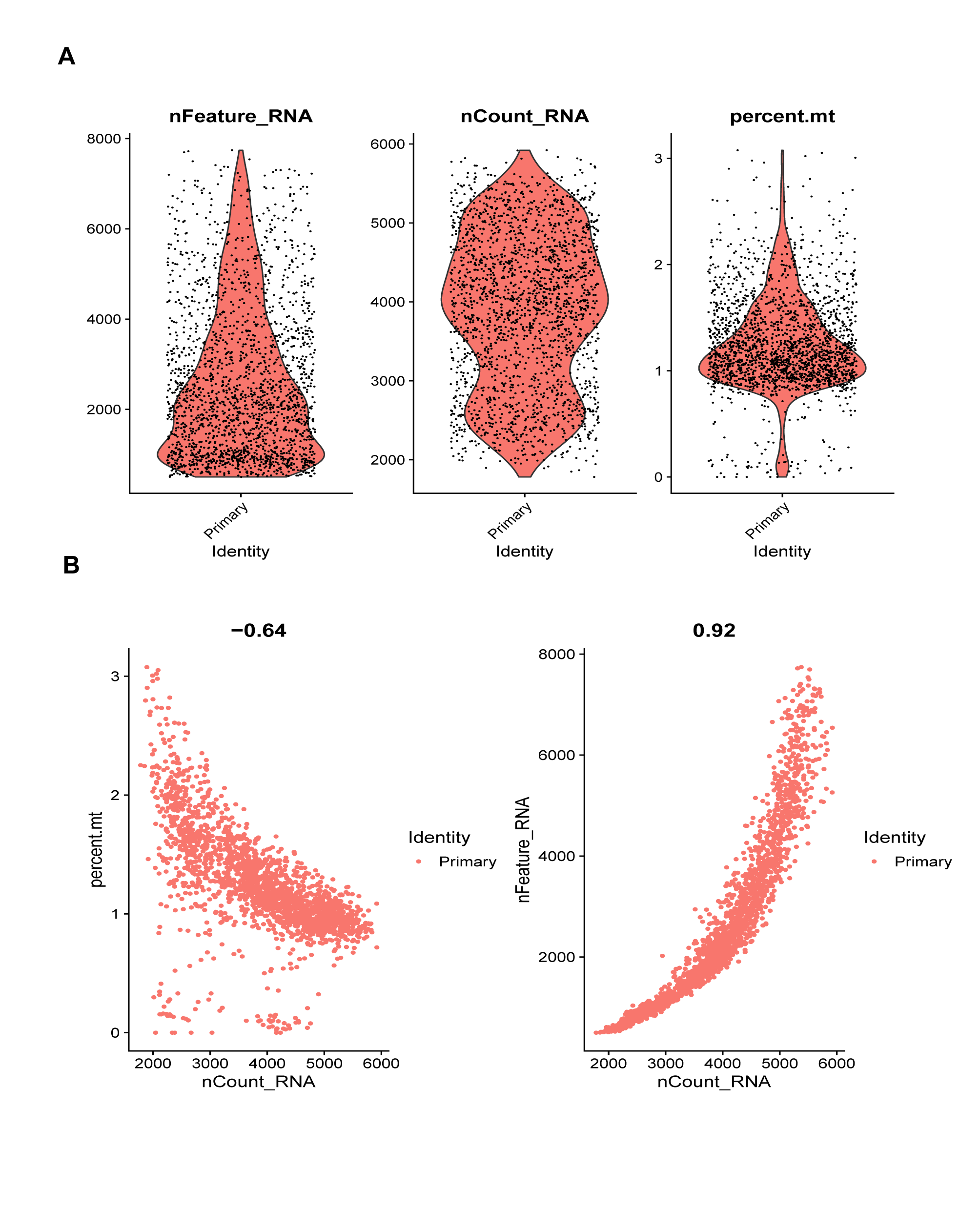

Supplement: Supplementary Figure 2 — Data filtration and quality control in data downloaded from GEO (GSE145137). (A) The number of RNA expressed in various cells and the percentage of mitochondrial genes. (B) The correlation of the counts of RNA expression and percentage of mitochondrial genes. The number of genes is positively related to the number of RNA. [file Image_2.tif]

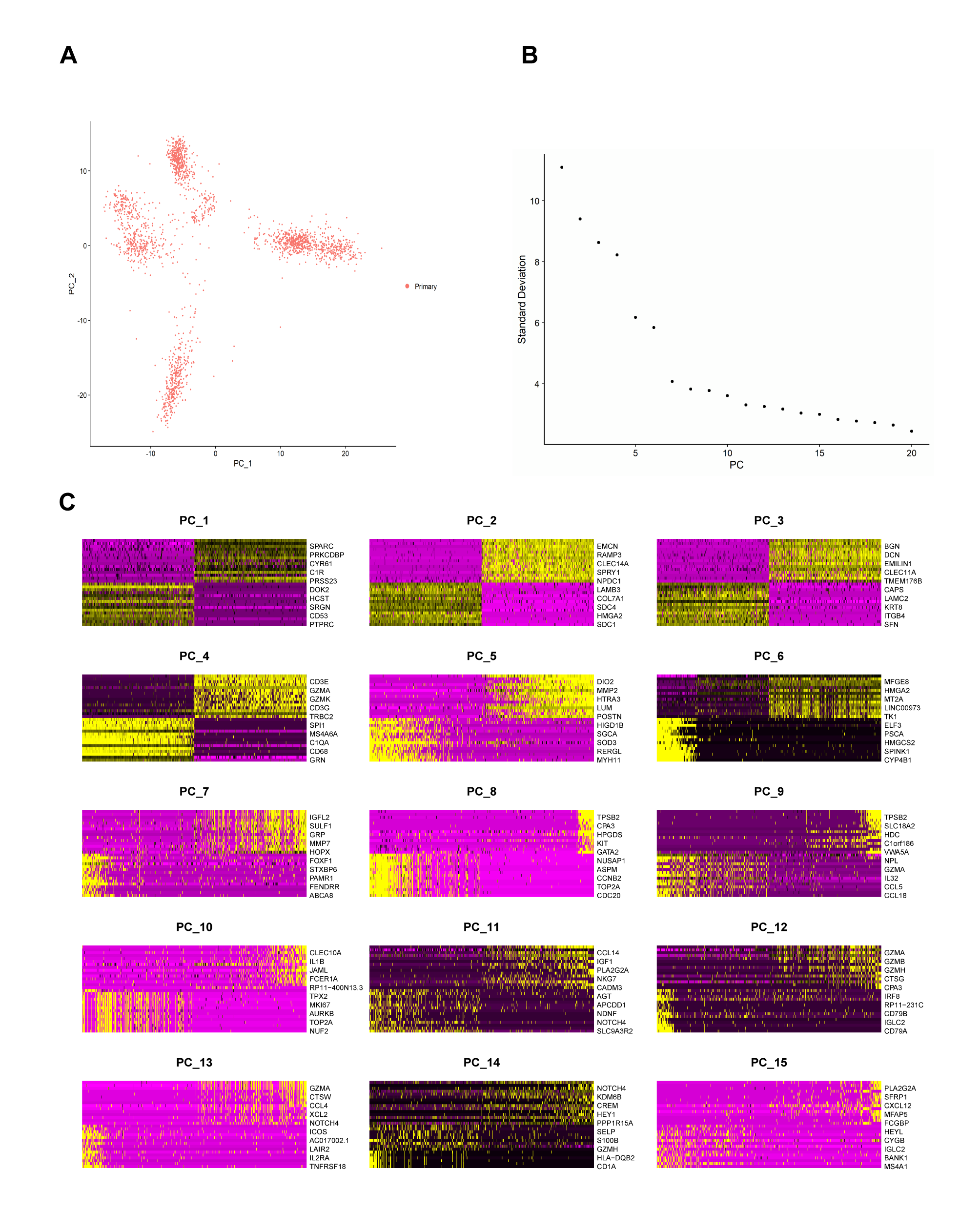

Supplement: Supplementary Figure 3 — Dimensionality reduction by PCA analysis (A) PCA results shown in scatter plot. (B) The ElbowPlot shows that results of PCA analysis. (C) The top 10 differentially expressed genes from PCA1 to PCA15. [file Image_3.tif]

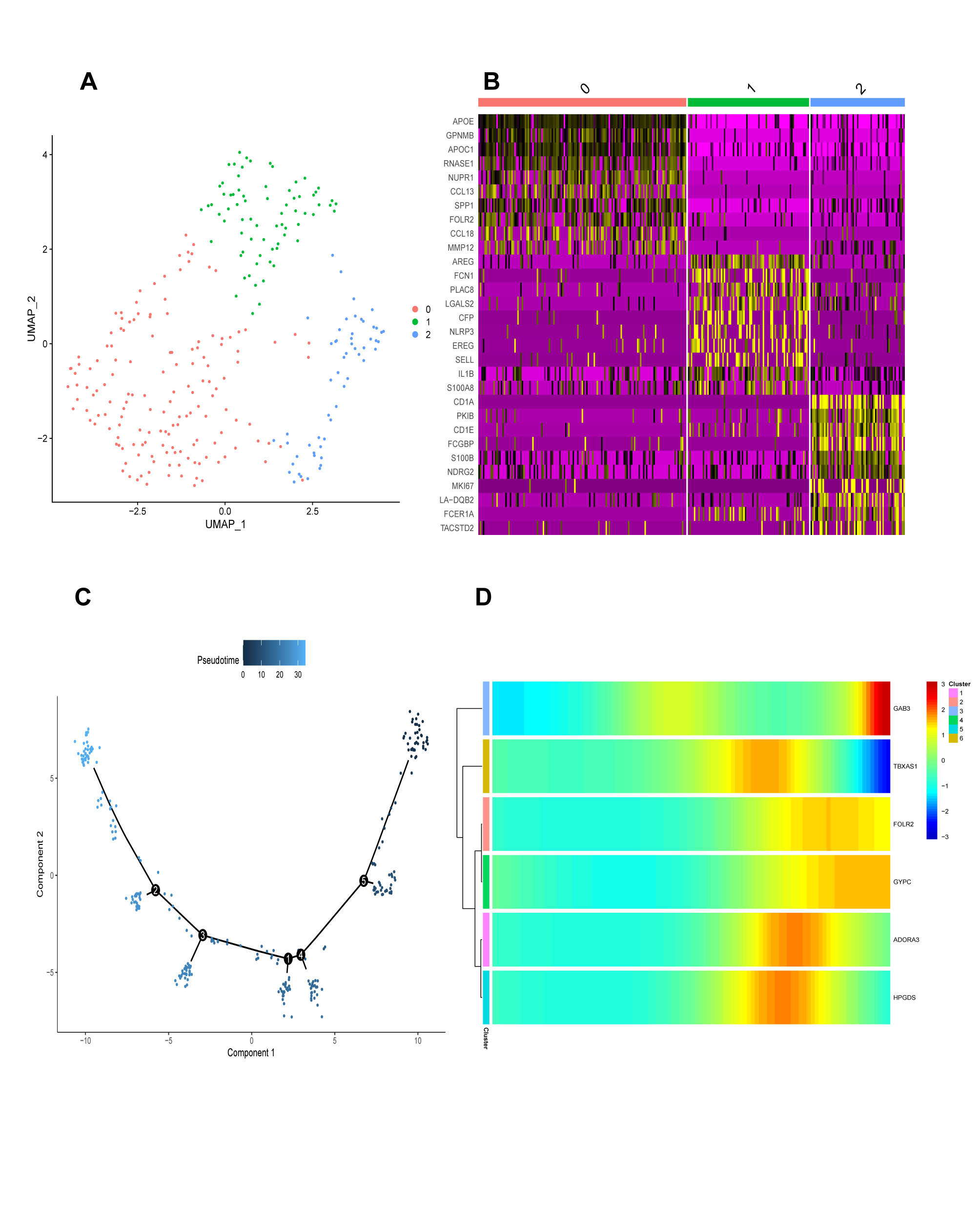

Supplement: Supplementary Figure 4 — Clustering and pseudo-chronological analysis of macrophages. (A) Umap plots of macrophage classification (B) The top 10 differentially expressed genes in three clusters. (C) Cell trajectory map established by pseudotime value. (D) The heatmap shows that the expression level of six genes in pseudo-chronological analysis. [file Image_4.tif]

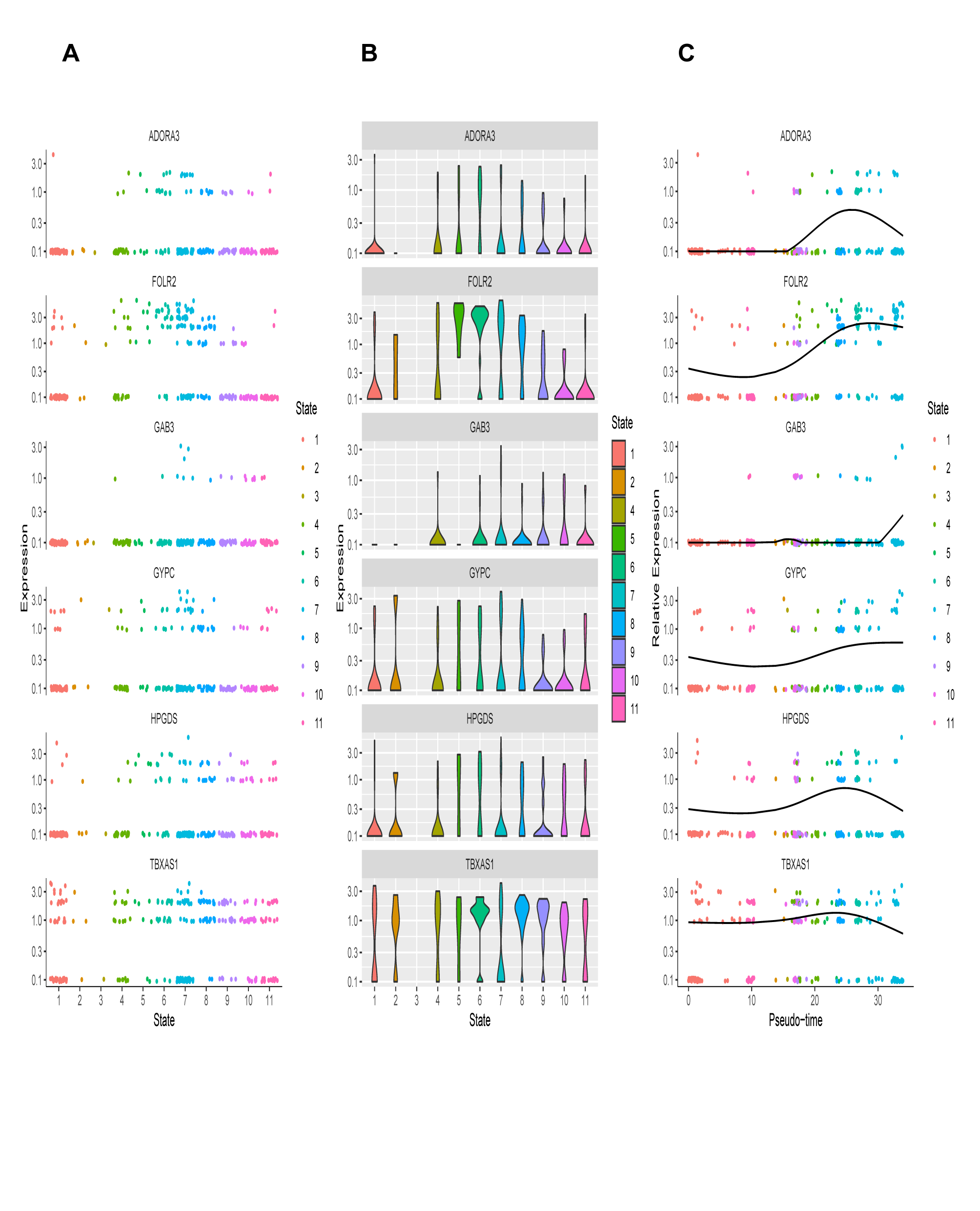

Supplement: Supplementary Figure 5 — The expression level of six genes with the changes in cell state. (A) The distribution of six genes in different cell state (B) The violin plot of the expression level of six genes in different cell state. (C) Line chart of the expression of six gene in different cell state. [file Image_5.tif]

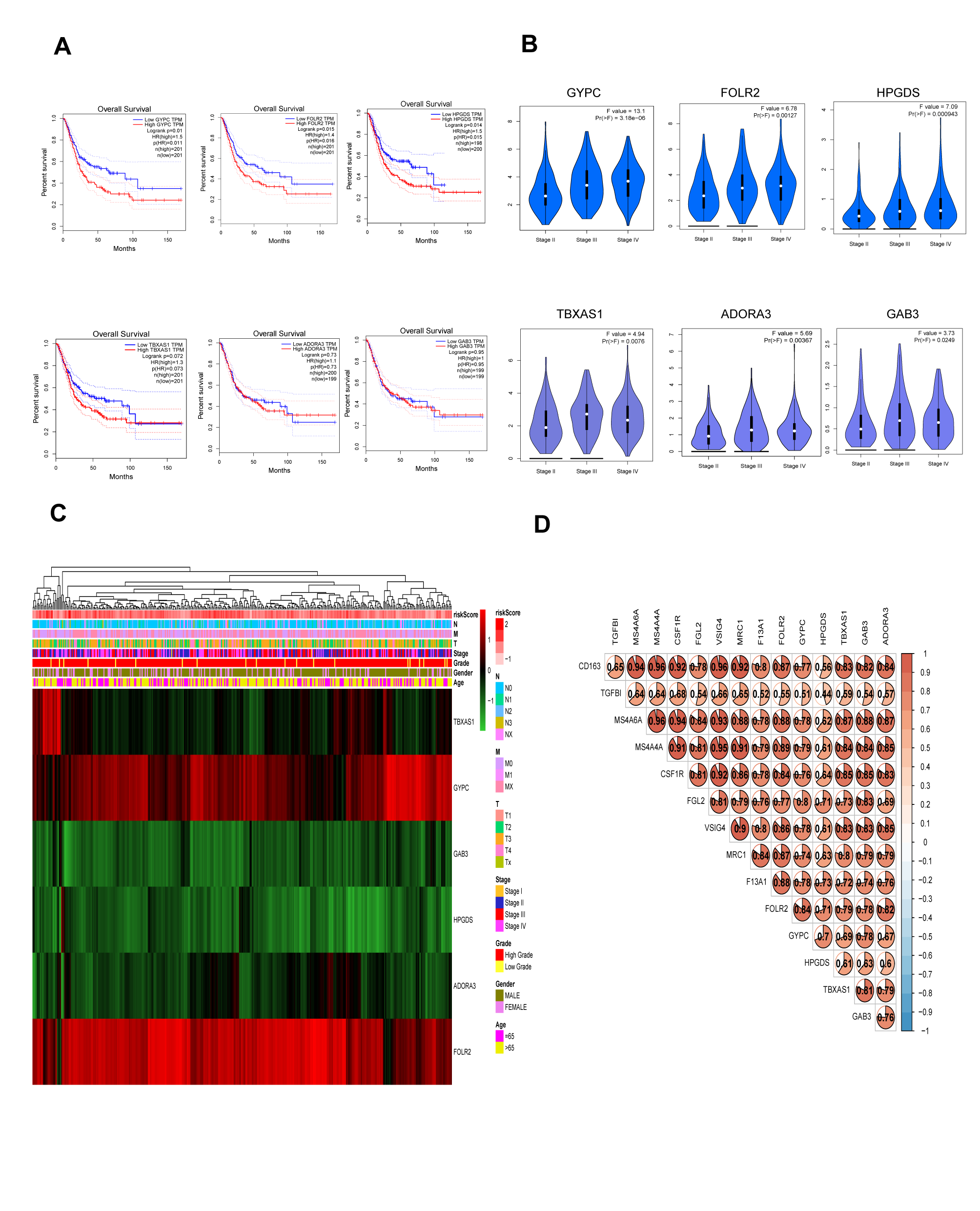

Supplement: Supplementary Figure 6 — The correlation between the expression level of six genes and clinical features. (A) The Kaplan Merier survival curve of TCGA-patients based on expression level of six genes. (B) The correlation between tumor stage and the expression level of six genes. (C) The heatmap shows the correlation between the expression of six gene and clinical features. (D) Heatmap shows that the correlation between common M2 biomakers and six gene signature. [file Image_6.tif]

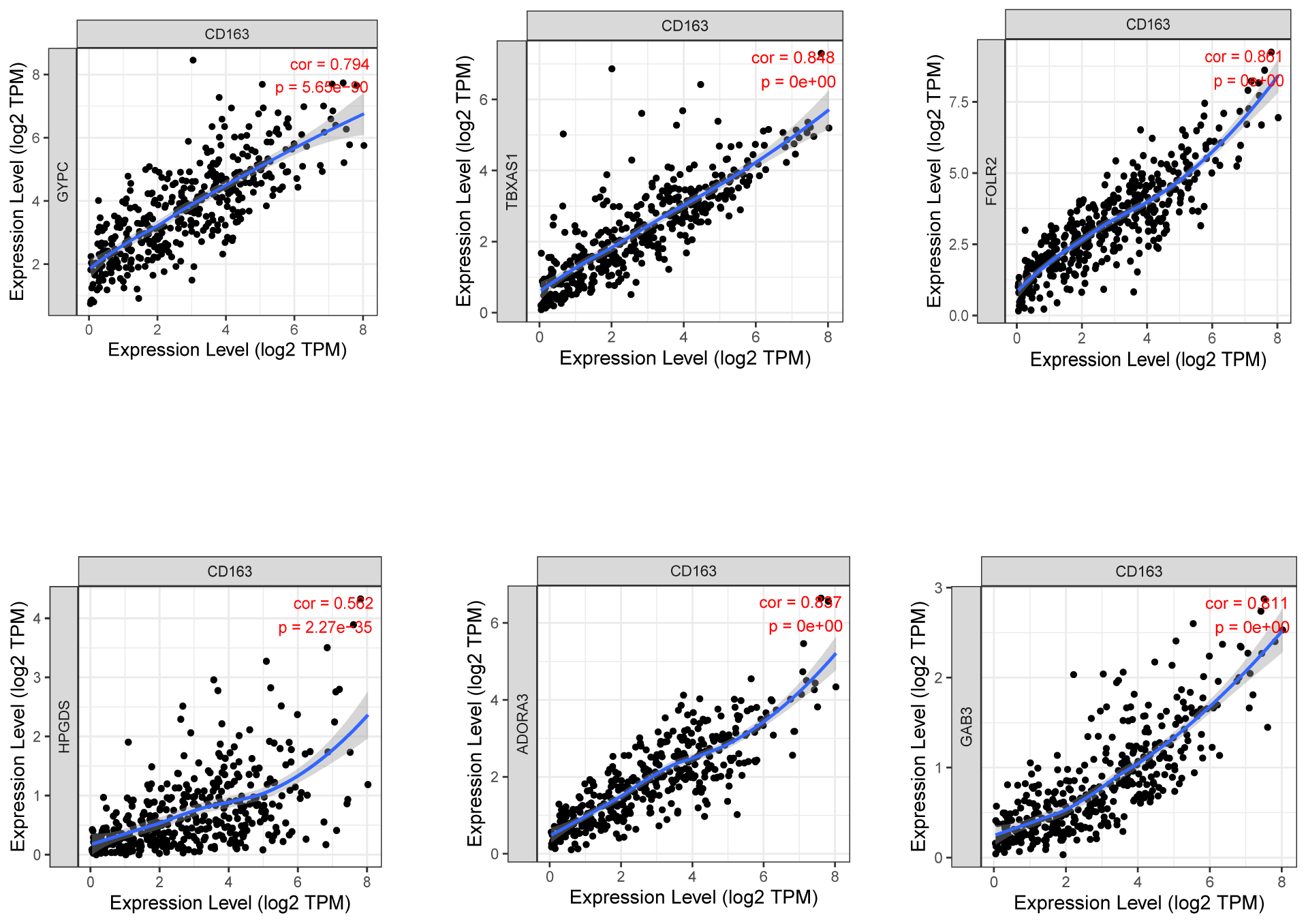

Supplement: Supplementary Figure 7 — The correlation between the expression level of six genes and the expression level of CD163. [file Image_7.tif]
